# Tranquilizing and Allaying Excitement Needling Method Affects BDNF and SYP Expression in Hippocampus

**DOI:** 10.1155/2017/8215949

**Published:** 2017-07-06

**Authors:** Peng Zheng, Xiaohong Xu, Hongyan Zhao, Tingting Lv, Bailin Song, Fuchun Wang

**Affiliations:** ^1^Jilin Provincial Hospital of Traditional Chinese Medicine, Changchun University of Chinese Medicine, Changchun 130021, China; ^2^Changchun University of Chinese Medicine, Changchun 130117, China; ^3^Institute of Basic Theory of Chinese Medicine, China Academy of Chinese Medical Science, Beijing 100700, China; ^4^Shanghai University of Traditional Chinese Medicine, Shanghai 201203, China

## Abstract

Sleep disorder is a state of sleep loss caused by various reasons, which leads to a series of changes, such as emotion, learning and memory, and immune function. “Tranquilizing and allaying excitement” was widely used in clinical treatment of insomnia; however, the mechanism was still not very clear. We randomly divided rats into three groups: control group, sleep deprivation group, and acupuncture treatment group. We observed BDNF and SYP expression in hippocampus in these three groups. Both protein contents and mRNA contents of BDNF and SYP were measured by western blot, immunohistochemistry, and RT-PCR analysis. The sleep deprivation model was established using modified multiple platform sleep deprivation method (MMPM). Our study explored the BDNF and SYP abnormality in hippocampus caused by sleep deprivation and “tranquilizing and allaying excitement” intervention regulated the abnormal expression of BDNF and SYP caused by sleep deprivation on the short run and the long run. Our study provided a molecular evidence that “tranquilizing and allaying excitement” treatment in rats with sleep disorder affects learning and memory ability.

## 1. Introduction

Acupuncture was a well-known traditional Chinese medicine [1–3]. Tranquilizing and allaying excitement needling method was acupuncture at a group of points to treat insomnia and it was often used in the clinical practice [4]. Although “tranquilizing and allaying excitement” acupuncture had significant effect on the treatment of insomnia in the clinic, the mechanism was not clear [5, 6]. It has been reported that sleep deprivation impaired learning and memory ability in both rats and mice [7–10], acupuncture had clinical effect on the treatment of insomnia [11], and our experiment was to discover whether acupuncture could improve the learning and memory ability impaired by sleep deprivation in rats. To study the effect of tranquilizing and allaying excitement needling method on learning and memory ability impaired by sleep deprivation, we used a modified multiple platform method (MMPM) to establish sleep deprivation rats model and then used tranquilizing and allaying excitement needling method to treat these sleep deprivation rats. As we all know, BDNF was an important regulator in many kinds of diseases [12], especially in learning and memory ability [13, 14]; learning and memory ability was seriously impaired when BDNF was deficient in the hippocampus [15]. We measured BDNF contents in the hippocampus in the sleep deprivation group and acupuncture group. Synaptophysin (SYP) is another key regulator of learning and memory ability in hippocampus [16]. The SYP contents in both the mRNA level and protein level in the hippocampus were measured in the sleep deprivation group and acupuncture group. We calculated IOD (Integrated Option Density) value of BDNF and SYP after histochemical staining in the hippocampus tissue. We were trying to figure out the expression level change of BDNF and SYP in the hippocampus after sleep deprivation and the expression level of BDNF and SYP after tranquilizing and allaying excitement needling method treatment of sleep deprivation. The effect of tranquilizing and allaying excitement needling method treatment on the expression of BDNF and SYP in the sleep deprivation was a critical evidence that tranquilizing and allaying excitement needling method improved learning and memory ability impaired by insomnia and it gave us some evidence in the mechanism of clinical treatment of insomnia by tranquilizing and allaying excitement needling method.

## 2. Materials and Methods

### 2.1. Experimental Animal

All experimental procedures were approved by the local ethics committee at Changchun Chinese Medicine University and were conducted in line with the Care and Use of Laboratory Animals published by the US National Institutes of Health (Publication, 8th Edition, 2011). Sixty adult Wistar rats weighting 180–220 g were purchased from experiment animal center of Jilin University (Jilin, China). 60 Wistar rats were randomly divided into three groups: control group, sleep deprivation group, and acupuncture treatment group. Each group is randomly divided into another two subgroups: the 24 h subgroup and 96 h subgroup, each subgroup containing 10 rats. All rats were fed under the same condition for 2 weeks and adapted in the platform for a week before the experiment, 1 h each day. We used the modified multiple platform method (MMPM) to establish the sleep deprivation rats model. The acupuncture treatment group was initiated one week before the experiment and stopped at the end of the experiment. The acupuncture acupoint was Shenmen, Sishencong, and Sanyinjiao; the stimulation amount was stabbing Shenmen 1 mm, stabbing Sanyinjiao 5 mm, and stabbing oblique Sishencong 2 mm, with normal reinforcement and normal reduction, retaining needle for 40 min. The controlled group was fed as normal without any treatment. Then the rat brains were extracted. Some of the rat brains were used to isolate the hippocampus to detect the BDNF mRNA and SYP mRNA contents by RT-PCR experiment, some of the rat brains were used to detect BDNF and SYP protein contents by western blot, and others were rapidly fixed in 4% paraformaldehyde to prepare for paraffin section and immunostaining in order to detect the BDNF and SYP proteins contents.

### 2.2. The Sleep Deprivation Rat Model

The rats adapted in the platform for 1 h per day for a week before the sleep deprivation experiment. The modified multiple platform method (MMPM) was used which was one of the most widely applied sleep deprivation rat models to establish the sleep deprivation model. The rat cages were of size 110 cm × 60 cm × 40 cm. There are 6 platforms totally; the distance between the nearest two platforms was 15 cm. Diameters and heights of the platforms were 6.5 cm and 8 cm, respectively. All these platforms were surrounded with water and they were only 1.0 cm above water. The temperature of water was 22°C and the temperature of room was maintained at 22–24°C. In the conscious state, a rat could feed and drink freely in the platforms. However, when a rat reached the Rapid Eye-Movement Sleep (REMS) state, its body would lose balance as the muscle tense decreased and the rats would wake up when touching the water. In that case a rat could not enter REMS state when it was located in the platform. There were 24 h sleep deprivation and 96 h sleep deprivation rat models, respectively, in these experiments. A 40 w light kept lighting during 08:30–20:30 in a day.

### 2.3. Tranquilizing and Allaying Excitement Needling Method

Tranquilizing and allaying excitement needling method was acupuncture at a group of acupoints including Shenmen point, Sishencong point, and Sanyinjiao point. There were two ways of acupuncture entering into skin, one of which is straight stabbing acupuncture and the other is oblique acupuncture. Both ways of acupuncture entering into skin and the time of acupuncture retaining in the skin were factors in the effect of treatment. In this experiment we kept the acupuncture retaining in the skin for 40 min. The acupuncture group was acupuncture at Sishencong (EX-HN 1), Shenmen (HT 7), and Sanyinjiao (SP 6) with tranquilizing and allaying excitement needling method.

### 2.4. Western Blot

BDNF and SYP protein were extracted from the hippocampus. Proteins were separated by electrophoresis on SDS-polyacrylamide gels and transferred moist to nitrocellulose filter membranes. Membranes were incubated with anti-BDNF antibody (A1307, 1 : 1000, Abclonal, China) and anti-SYP antibody (ab64581, 1 : 1000, Abcam, USA) overnight at 4°C. After washing, the membrane was incubated with IRDye secondary antibodies (LI-COR) for 1 h. The images were captured by the Odyssey CLx Near-Infrared Imaging System (LI-COR Biosciences, Lincoln, NE, USA). Western blot bands were quantified by measuring intensity in each group using Odyssey CLx version 2.1 with anti-*β*-actin antibody (TA-09, 1 : 1000, ZSGB-Bio, China) as an internal control.

### 2.5. RNA Extraction and Real-Time PCR

Total BDNF RNA and SYP RNA were extracted from hippocampus tissue of rats using Trizol reagent (Invitrogen, USA) according to protocols. RNA quantity was assessed using the NanoDrop™ 8000 spectrophotometer (Thermo Scientific, France). cDNA was synthesized using reverse transcriptase kit (Roche, USA). Sequences of gene-specific PCR primers (Shanghai Generay Biotech Co. Ltd., China) used were as follows: BDNF mRNA: upstream: 5′-AGCTGAGCGTGTGTGACAGT-3′; downstream: 5′-GCCAATTCTCTTTTTGCTATCCA-3′; SYP mRNA: upstream: 5′-TCTGGTGATGTTTTACGCTAC-3′; downstream 5′-AATCCTCAGGCTTAACTTGC-3′; *β*-actin mRNA: upstream: 5′-TATCGGACGCCTTGGTTAC-3′; downstream 5′-CTGTGCCGTTGAACTTGC-3′.

The RT-PCR of BDNF mRNA and SYP mRNA was performed in 20 *μ*l volumes with SYBR Green PCR Master Mix (Roche, USA) at 95°C for 10 min and 40 cycles at 95°C for 15 s, 60°C for 30 s, and 72°C for 30 s, using LightCycler 480 (Roche, USA).

### 2.6. HE Staining

Animals were anesthetized with pentobarbital and perfused transcardially with saline and then with phosphate-buffered 10% formalin. Brains were removed and processed for paraffin embedding. Three-micrometer coronal sections were cut at the level of the dorsal hippocampus and then used for hematoxylin and eosin (HE) staining.

### 2.7. Immunohistochemistry

Anti-rat BDNF antibody [rabbit IgG ab182199] and anti-rat SYP antibody [rabbit IgG, ab8049] were purchased from Bay Bioscience (Hyogo, Japan) and Wako Pure Chemical (Osaka, Japan), respectively. The paraffinized sections were blocked from endogenous peroxidase activity by incubation in distilled water containing 3% hydrogen peroxide for 5 min. Antigen retrieval was performed, using a 0.01 M citrate buffer (pH 6.0) for both anti-rat BDNF antibody and anti-rat SYP antibodies by the Pascal heat-induced target retrieval system (DAKO Japan, Tokyo, Japan). Nonspecific binding sites were blocked in 0.01 M phosphate-buffered saline (PBS), pH 7.4, containing 2% bovine serum albumin (BSA; Wako Pure Chemical) for 60 min. Both anti-rat BDNF and anti-rat SYP antibodies used at a dilution of 1 : 100 in 2% BSA. PBS were added on the slides and incubated overnight at 4°C. BDNF and SYP were detected with a biotinylated anti-rabbit IgG (1 : 1000) for 30 min, followed by incubation with avidin-coupled peroxidase (Vectastain ABC Kit, Vector Laboratories, Burlingame, USA) for 30 min.

### 2.8. Statistical Analysis

All data are expressed as mean ± SEM and analyzed by Image-Pro Plus 6.0 system. Two-group comparisons were performed by Student's *t*-test. Multiple-group comparisons were carried out using one-way ANOVA followed by Dunnett's *t*-test. A two-tailed *p* < 0.05 was considered to be significant.

## 3. Results

### 3.1. Tranquilizing and Allaying Excitement Needling Method Affects the BDNF and SYP Expression

The concentration of the serum NO was measured by extracting the eyeball blood in 24 h and 96 h after sleep deprivation. The NO contents of control group were maintained in a relatively steady level which were much lower than the sleep deprivation group. The NO contents in the sleep deprivation group are gradually increased along with the sleep deprivation time (*p* < 0.05). The NO contents of acupuncture treatment group are higher than the control group but lower than the sleep deprivation group and are also increased along with the sleep deprivation time (*p* < 0.05). Both the relative expression level of BDNF mRNA which was measured by RT-PCR detection and that of the BDNF protein contents which was measured by western blot indicated that the BDNF contents of the control group were maintained in a relatively normal state; however, the BDNF expression level in sleep deprivation group was gradually decreased along with sleep deprivation time, which was significantly higher than the control group at 24 h hours but lower than the control group at 96 h (*p* < 0.05). The SYP expression level in the control group stayed at a relatively high level; however, the SYP expression level in the acupuncture treatment group gradually decreased according to the sleep deprivation time, which are significantly lower than the control group (*p* < 0.05). The relative expression level of SYP mRNA is also gradually decreased according to the sleep deprivation time and was significantly higher than the control group but lower than the acupuncture group (*p* < 0.05).

### 3.2. BDNF mRNA and SYP mRNA Expression in Rat Hippocampus

#### 3.2.1. The Total RNA Extraction in Rat Hippocampus

 Regarding the total RNA extracted from rat hippocampus, agarose electrophoresis detection indicates that the strip of 28S and 18S RNA was obvious. The OD260/OD280 were between 1.8 and 2.2 which demonstrated that RNA was not degenerated.

#### 3.2.2. BDNF mRNA Expression in Rat Hippocampus

**Figure 1:**
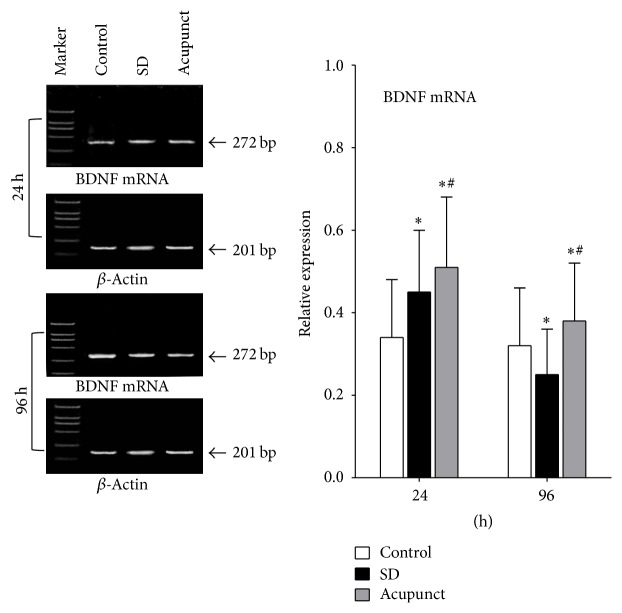
Optical density ratio of BDNF mRNA in rats hippocampus tissue. ^*∗*^Compared to the control group, *p* < 0.05; ^#^compared to the sleep deprivation group, *p* < 0.05.

We used 201 bp *β*-actin mRNA for gray value comparison; the BDNF mRNA expression contents are measured by BDNF mRNA/*β*-actin mRNA. The relative expression level of BDNF mRNA is maintained at the same level in control rats; the BDNF mRNA expression level in acupuncture rats gradually decreased along with the sleep deprivation time, which is significantly higher than the control group at 24 h and significantly lower than the control group at 96 h (*p* < 0.05) (Figure 1). The relative mRNA expression level in acupuncture treatment group gradually decreased along with the sleep deprivation time and was significantly higher than the control group and sleep deprivation group (*p* < 0.05).

#### 3.2.3. SYP mRNA Expression in Rat Hippocampus

**Figure 2:**
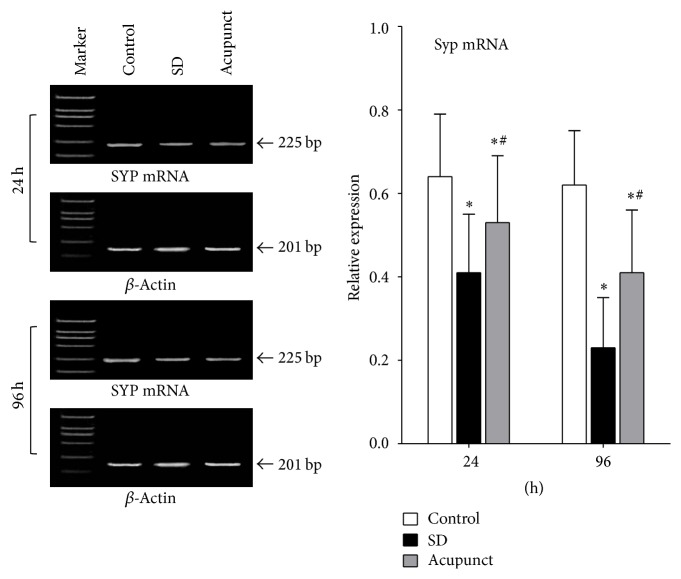
Optical density ratio of SYP mRNA in rats' hippocampus tissue. ^*∗*^Compared to the control group, *p* < 0.05; ^#^compared to the sleep deprivation group, *p* < 0.05.

The ratio of SYP mRNA/*β*-actin mRNA was used as a relative expression level of SYP mRNA, which indicated the expression level change of SYP mRNA; *β*-actin mRNA was used as a control. According to the figure, the SYP mRNA relative expression level in control rats was maintained at the same level. The mRNA relative expression level in sleep deprivation rats gradually decreased, which was significantly lower than the control group (*p* < 0.05) (Figure 2). The mRNA relative expression level in acupuncture rats gradually decreased, which was significantly lower than the control group and higher than the sleep deprivation group (*p* < 0.05).

### 3.3. BDNF and SYP Protein Expression in Rat Hippocampus

#### 3.3.1. Western Blot Analysis of the BDNF Protein Expression

**Figure 3:**
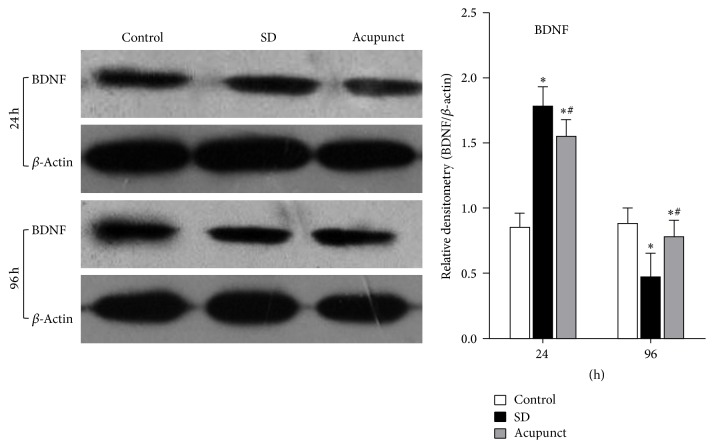
Western blot detected of BDNF in rats hippocampus tissue. ^*∗*^Compared to the control group, *p* < 0.05; ^#^compared to the sleep deprivation group, *p* < 0.05.

As we can see in the picture of the BDNF expression in rat hippocampus (Figure 3), using *β*-actin as a control, the band was under a gradation analysis, taking the ratio of BDNF/*β*-actin as a relative expression level of BDNF to measure the BDNF expression level change. According to the result of western blot, the BDNF expression level was maintained at the same level in control rats, while BDNF expression in acupuncture group quickly increased after 24 h of sleep deprivation, which was significantly higher than the control group (*p* < 0.05) and, after 96 h of sleep deprivation, the BDNF expression level decreased, which was significantly lower than the control group (*p* < 0.05). The BDNF expression level in acupuncture treatment group was significantly increasing and higher than the control group after 24 h (*p* < 0.05) and was decreased after 96 h, which is significantly lower than the control group and higher than the acupuncture treatment group (*p* < 0.05).

#### 3.3.2. Western Blot Analysis of the SYP Protein Expression

**Figure 4:**
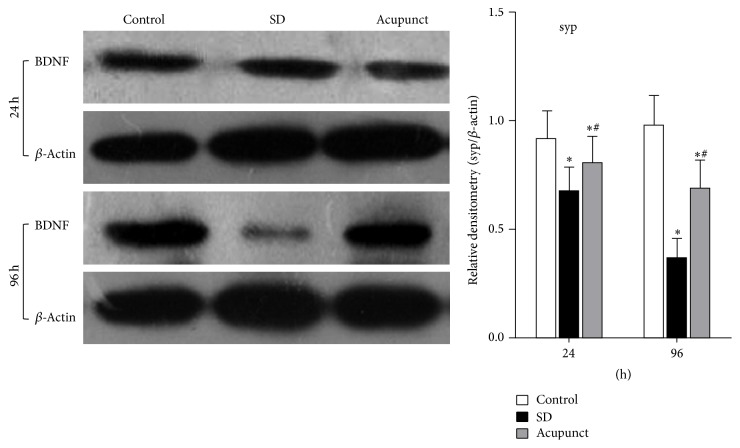
Optical density ratio of SYP in rats hippocampus tissue. ^*∗*^Compared to the control group, *p* < 0.05; ^#^compared to the sleep deprivation group, *p* < 0.05. 1: control group; 2: sleep deprivation group; 3: acupuncture treatment group.

SYP protein expression level is shown in the figure (Figure 4). we took a gradation analysis of the band; *β*-actin was used as a control. The SYP relative expression level is measured by the ratio of SYP/*β*-actin to calculate the expression level change of SYP. After the statistics analysis of the band gradation, we can conclude that the SYP expression level in the control rats was maintained at the same level, while the SYP expression level in the sleep deprivation group gradually decreased along with the sleep deprivation time, which was significantly higher than the control group (*p* < 0.05). The SYP level in the acupuncture treatment group also gradually decreased along with the sleep deprivation time, which was significantly lower than the control group but higher than the sleep deprivation group (*p* < 0.05).

### 3.4. Immunohistochemistry Analysis

#### 3.4.1. HE Staining

**Figure 5:**
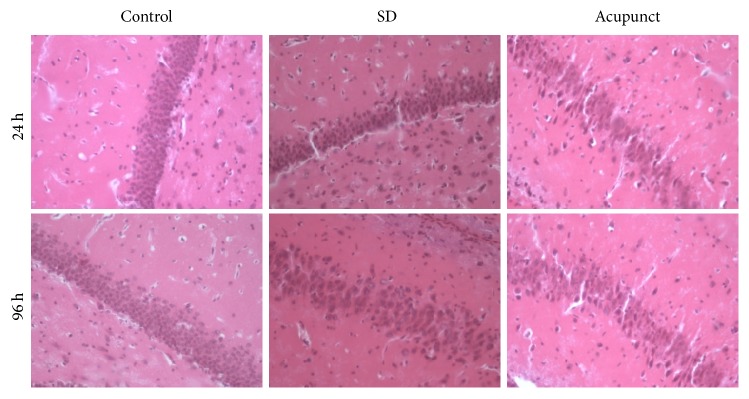
Rats' hippocampus CA1 HE stain slide of each group (×400).

We can figure out from the figure that the HE stained neurons in the CA1 of rat of hippocampus were clearly visible. The neurons were arranged densely (Figure 5). There was not any inflammatory cell invasion. The cell nucleus was intact and there was no apoptosis phenomenon both 24 h and 96 h after sleep deprivation. The CA1 neurons of hippocampus in sleep deprivation group and acupuncture treatment group were not significantly different from the control group.

#### 3.4.2. Immunohistochemistry Analysis of BDNF Expression in Rat Hippocampus

**Figure 6:**
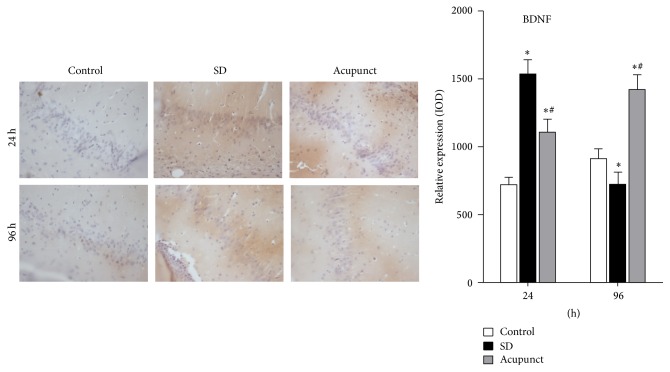
Hippocampus CA1 immunohistochemical stain slide of BDNF (×400). ^*∗*^Compared to the control group, *p* < 0.05; ^#^compared to the sleep deprivation group, *p* < 0.05.

As we can see from the figure, there were BDNF positive areas which were sepia in the CA1 region of rat hippocampus tissue. The cell shape is clearly visible. The positive area of BDNF in the sleep deprivation group and acupuncture treatment group was significantly larger than the control group. We analyze the IOD value of BDNF positive expression area using Image-Pro Plus 6.0. After the statistics analysis of the IOD values, we concluded that the BDNF expression level in the control group was maintained at the same level. The BDNF positive area in the sleep deprivation group was significantly increased after 24 h of sleep deprivation and the IOD values significantly increased (*p* < 0.05) (Figure 6); both the BDNF positive area and IOD values were significantly decreased after 96 h of sleep deprivation (*p* < 0.05). The BDNF positive area and IOD values were significantly increased in the acupuncture treatment group after 24 h of sleep deprivation, which were significantly higher than the control group (*p* < 0.05). The BDNF positive area colored sepia were significantly decreased after 96 h in the acupuncture treatment group, which were lower than the control group but higher than the sleep deprivation group (*p* < 0.05).

#### 3.4.3. Immunohistochemistry Analysis of SYP Expression in Rat Hippocampus

**Figure 7:**
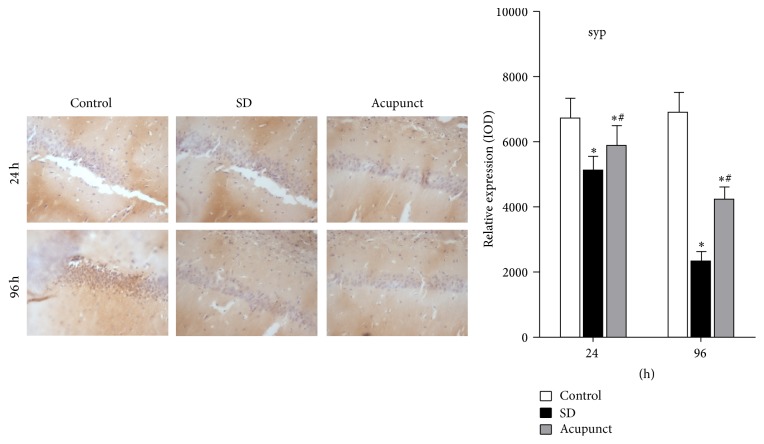
Hippocampus CA1 immunohistochemical stain slide of SYP (×400). ^*∗*^Compared to the control group, *p* < 0.05; ^#^compared to the sleep deprivation group, *p* < 0.05.

As it can be seen from the figure, the CA1 neurons of rat hippocampus demonstrated a sepia BDNF positive area after immunohistochemistry. The cell shapes could clearly be seen. We calculate the IOD values of the BDNF positive area using the software Image-Pro Plus 6.0. The SYP expression level in the control group stayed at the same level. However, the sepia area which showed SYP positive expression in the sleep deprivation was gradually decreasing as the sleep deprivation time went on; the IOD values were significantly lower than the control group (*p* < 0.05) (Figure 7). The SYP positive area in the acupuncture treatment group after sleep deprivation was gradually deceasing according to the deprivation time and the IOD values gradually decreased, which were lower than the control group and higher than the sleep deprivation group (*p* < 0.05).

## 4. Discussion

The synaptic plasticity including synaptic transmission efficiency and synaptic formation is the neurobiological fundamental of learning and memory. Selectively destroyed hippocampus impaired learning and memory ability; patients whose CA region of hippocampus was specifically impaired demonstrated anterograde amnesia behavior [17]. LTP is a functional indicator of synaptic plasticity and is closely related to BDNF and SYP expression in hippocampus. Synaptic activity in hippocampus facilitated rise of BDNF in hippocampus which regulated LTP. It has been reported that the stimulus pattern which had induced LTP production increased BDNF mRNA expression in CA1 of hippocampus [18]. Research by Hairston indicated that BDNF expression was abnormal in rats with sleep disorder [19]. The BDNF level was slightly increased after 6 h of sleep deprivation and was significantly dropped after 96 h of sleep deprivation [19], which was coinciding with our experiment. At the early stage of sleep deprivation (within 24 h), organic had a feedback regulation against the drop of BDNF induced by sleep deprivation; as a result the BDNF expression level had a slight increase. At the late stage of sleep deprivation, the feedback regulation of organic was dysfunctional, so the BDNF expression level significantly dropped. After acupuncture in rats with 24 h or 94 h sleep deprivation, we measured the BDNF expression level in CA1 of hippocampus. BDNF in 24 h sleep deprivation group was downregulated by acupuncture and BDNF in 96 h sleep deprivation group was upregulated by acupuncture. The results indicated that acupuncture had a positive effect on abnormal BDNF expression of either 24 h or 96 h sleep deprivation rats. BDNF regulates synaptic activity and synaptic activity has been well known; the regulation function of acupuncture on BDNF partly illustrated the pathway of tranquilizing and allaying excitement needling method treatment in patients with sleep disorder.

SYP is downstream of BDNF and tyrosine kinase cascade reaction; BDNF increases glutamic acid release by strength MAPK dependent synaptic protein I phosphorylation. The induction of LTP is link-coupled with SYP synthesis. SYP is closely related to synaptic plasticity [20]. We had observed the BDNF gene and protein expression level in CA region of hippocampus; to further assess treatment effect of acupuncture on rat with sleep deprivation, we observed the SYP expression level. SYP is a specific membrane protein of synaptic vehicles; the decrease of SYP impaired transport capacity of synaptic vehicles, which finally reduced synaptic transmission, message process, and message storage [21]. The expression level of SYP was positively related to learning and memory ability. It has been proved that the hippocampus structure in AD patients was significantly smaller than in normal subjects [22]. The level of cognitive disorder in AD patients was related to immune activity of synaptophysin [23]. Our study demonstrated that SYP expression level in CA region dropped along with time and acupuncture upregulated SYP expression level in CA1 after either 24 h or 96 h sleep deprivation, which showed that acupuncture upregulated the abnormal expression of SYP after either 24 h or 96 h sleep deprivation. The regulation function of acupuncture on SYP MRNA level coincided with that on SYP protein level. In our study, we observed the regulation function of acupuncture on BDNF and SYP in CA region of hippocampus; however, we did not find out what pathway the acupuncture regulated on the expression of BDNF and SYP. We will validate our conclusion by further detecting the effect of acupuncture on LTP.
